# Supplementary material for: PRETIE-Q Spanish Version and Affective-Functional Responses to Age-Based Strength Training in Older Women: An Exploratory Study
Source: Healthcare (Basel). 2025 Nov 21;13(23):3000. doi: 10.3390/healthcare13233000 (PMC12692431; doi:10.3390/healthcare13233000)
Supplement: Supplementary file 1 [file healthcare-13-03000-s001.zip › healthcare-3950133-supplementary.pdf]

Supplementary Material

Table S1: Cuestionario de Preferencia y Tolerancia a la Intensidad del Ejercicio (versión en español)

N°	Por favor, lea cada una de las siguientes afirmaciones y, a continuación, utilice la escala de respuestas de la derecha para indicar si está de acuerdo o en desacuerdo con ellas. No hay respuestas correctas o incorrectas. Conteste rápidamente y marque la respuesta que mejor describa lo que cree y como se siente. Asegúrese de responder a todas las preguntas.	*Estoy totalmente en desacuerdo*	*Estoy en desacuerdo*	*No estoy ni en acuerdo ni en desacuerdo*	*Estoy de acuerdo*	*Estoy totalmente de acuerdo*
1	Sentir cansancio durante el ejercicio es mi señal para bajar el ritmo o detenerme.	**1**	**2**	**3**	**4**	**5**
2	Prefiero entrenar a baja intensidad por un tiempo prolongado antes que a alta intensidad por un tiempo corto.	**1**	**2**	**3**	**4**	**5**
3	Durante el ejercicio, si mis músculos comienzan a arder demasiado o si me cuesta mucho respirar, es momento de aflojar.	**1**	**2**	**3**	**4**	**5**
4	Prefiero ir despacio en mi entrenamiento, aunque signifique tomarme más tiempo.	**1**	**2**	**3**	**4**	**5**
5	Cuando hago ejercicio, trato de seguir incluso después de sentirme agotado(a).	**1**	**2**	**3**	**4**	**5**
6	Prefiero un entrenamiento corto e intenso antes que uno largo y de baja intensidad.	**1**	**2**	**3**	**4**	**5**
7	Bloqueo la sensación de fatiga cuando estoy ejercitándome.	**1**	**2**	**3**	**4**	**5**
8	Cuando entreno, normalmente prefiero un ritmo lento y constante.	**1**	**2**	**3**	**4**	**5**
9	Prefiero bajar el ritmo o parar cuando el entrenamiento se pone demasiado difícil.	**1**	**2**	**3**	**4**	**5**
10	Hacer ejercicio a baja intensidad no me atrae para nada.	**1**	**2**	**3**	**4**	**5**
11	La fatiga es lo último que influye cuando decido detener un entrenamiento; tengo una meta y paro solo al alcanzarla.	**1**	**2**	**3**	**4**	**5**
12	Al ejercitarme, prefiero actividades de ritmo lento que no requieran tanto esfuerzo.	**1**	**2**	**3**	**4**	**5**
13	Cuando mis músculos empiezan a arder durante el ejercicio, normalmente bajo un poco la intensidad.	**1**	**2**	**3**	**4**	**5**
14	Mientras más rápido y exigente es el entrenamiento, más agradable me resulta.	**1**	**2**	**3**	**4**	**5**
15	Siempre supero el dolor muscular y la fatiga cuando entreno.	**1**	**2**	**3**	**4**	**5**
16	El ejercicio de baja intensidad me resulta aburrido.	**1**	**2**	**3**	**4**	**5**
